# Computational and *in vitro* experimental analyses of the anti-COVID-19 potential of Mortaparib and Mortaparib^Plus^

**DOI:** 10.1042/BSR20212156

**Published:** 2021-10-14

**Authors:** Vipul Kumar, Anissa Nofita Sari, Hazna Noor Meidinna, Jaspreet Kaur Dhanjal, Chandru Subramani, Brohmomoy Basu, Sunil C. Kaul, Sudhanshu Vrati, Durai Sundar, Renu Wadhwa

**Affiliations:** 1 DAILAB, Department of Biochemical Engineering and Biotechnology, Indian Institute of Technology (IIT) Delhi, Hauz Khas, New Delhi 110016, India; 2 AIST-India DAILAB, DBT-AIST International Center for Translational and Environmental Research (DAICENTER), National Institute of Advanced Industrial Science and Technology (AIST), Tsukuba 3058565, Japan; 3 Department of Computational Biology, Indraprastha Institute of Information Technology Delhi, Okhla Industrial Estate, Phase III, New Delhi 110020, India; 4 Regional Centre for Biotechnology (RCB), Faridabad 121001, India

**Keywords:** Antiviral assays, cancer biology, computational biology, COVID-19, Mortaparib, SARS-CoV-2

## Abstract

Coronavirus disease 2019 (COVID-19) pandemic caused by severe acute respiratory syndrome coronavirus 2 (SARS-CoV-2) virus has become a global health emergency. Although new vaccines have been generated and being implicated, discovery and application of novel preventive and control measures are warranted. We aimed to identify compounds that may possess the potential to either block the entry of virus to host cells or attenuate its replication upon infection. Using host cell surface receptor expression (angiotensin-converting enzyme 2 (ACE2) and Transmembrane protease serine 2 (TMPRSS2)) analysis as an assay, we earlier screened several synthetic and natural compounds and identified candidates that showed ability to down-regulate their expression. Here, we report experimental and computational analyses of two small molecules, Mortaparib and Mortaparib^Plus^ that were initially identified as dual novel inhibitors of mortalin and PARP-1, for their activity against SARS-CoV-2. *In silico* analyses showed that Mortaparib^Plus^, but not Mortaparib, stably binds into the catalytic pocket of TMPRSS2. *In vitro* analysis of control and treated cells revealed that Mortaparib^Plus^ caused down-regulation of ACE2 and TMPRSS2; Mortaparib did not show any effect. Furthermore, computational analysis on SARS-CoV-2 main protease (M^pro^) that also predicted the inhibitory activity of Mortaparib^Plus^. However, cell-based antiviral drug screening assay showed 30–60% viral inhibition in cells treated with non-toxic doses of either Mortaparib^Plus^ or Mortaparib. The data suggest that these two closely related compounds possess multimodal anti-COVID-19 activities. Whereas Mortaparib^Plus^ works through direct interactions/effects on the host cell surface receptors (ACE2 and TMPRSS2) and the virus protein (M^pro^), Mortaparib involves independent mechanisms, elucidation of which warrants further studies.

## Introduction

The novel severe acute respiratory syndrome coronavirus 2 (SARS-CoV-2) is an RNA virus that belongs to *Coronaviradae* family. Its genetic material, a large positive-sense strand of RNA (∼30 kb), is protected by a layer of nucleocapsid, which comprises three major structural proteins *viz*., membrane (M) protein that protects genome and facilitates fusion and assembly of the virus, envelope (E) protein that forms an integral part of the viral membrane and plays a crucial role in the assembly and pathogenesis, and spike (S) protein that gives it a crown-like appearance and plays a key role in the fusion and entry of the virus in the host cell [1–9]. Coronaviruses are divided into four major genera (α, β, γ, and δ). Among these, the former two are known to infect mammals while the latter two infect birds. SARS-CoV-2 is a β-coronavirus [10–12]. The outbreak of novel coronavirus disease 2019 (COVID-19) in the Wuhan province of China by the SARS-CoV-2 was declared a global pandemic by the World Health Organization [13]. SARS-CoV-2 is extremely contagious, several folds more than its previous close relatives, SARS-CoV and MERS coronaviruses [14]. As of 10 September 2021, there are >223 million cases reported with >4.6 million deaths worldwide as recorded by WHO.

Based on the SARS-CoV-2 genome information and crystal structure of its important proteins, anti-COVID-19 drug discovery has been initiated using a variety of approaches. These mainly include (i) suppression of essential viral proteins/RNA, (ii) interference of viral entry/replication in the host cells, and (iii) direct killing of the virus [15,16]. In the current scenario that lacks established medical treatments for lethal COVID-19, prevention and mitigation of new infections is considered as the preferred approach [17]. Targeting either the host cell proteins that aid in viral infection and/or replication of virus inside the host cells is widely considered as reliable and promising interventional approach. Among several proteins implicated as potential targets, host cell membrane proteins; transmembrane protease serine 2 (TMPRSS2) and angiotensin-converting enzyme 2 (ACE2) play a central role in viral infection and entry [5,18,19]. The glycosylated spike protein of SARS-CoV-2 initiates the infection by binding to the cell surface receptor ACE2 of the host cell [5,18], following which it gets cleaved and activated by host cell membrane protein TMPRSS2 [5,19,20]. After the attachment, fusion, and entry of the virus into the host cell, the genetic material of the virus (positive-sense ssRNA) gets translated into various polyproteins using host cell translation machinery. However, many of the translated polypeptides exist in an inactive state and are converted into functional state upon cleavage by other SARS-CoV-2 proteases. Among these translated polyproteins, a crucial viral protease enzyme called main protease (M^pro^) has been shown to activate itself and cleave other SARS-CoV-2 polyproteins [21–23] that are essential for virus replication, assembly, and transmission. SARS-CoV-2 has been reported to possess higher binding affinity to ACE2 as compared with SARS-CoV [24] suggesting ACE2 as a potential target. However, ACE2 receptor protein is involved in host cells’ physiological functions, thus, its strong suppression could be counterproductive [25–29]. TMPRSS2, on the other hand, has been shown to be involved in pathological processes [25,30]. M^pro^ is coded by the SARS-CoV-2 using the host cell resources and is pathologically highly relevant. In view of these, ACE2, TMPRSS2, and M^pro^ have been considered as potential drug targets for COVID-19 therapy.

## Materials and methods

### Compounds and cell culture

Human esophageal squamous cell carcinoma (T.Tn) were procured from the Japanese Collection of Research Bioresources Cell Bank (JCRB), Japan. Cells were cultured in Dulbecco’s modified Eagle’s medium (DMEM) (Fujifilm WAKO Pure Chemical Corporation, Osaka, Japan) supplemented with 5% fetal bovine serum (Thermo Fisher Scientific, Japan) and 1% penicillin/streptomycin (Invitrogen, Carlsbad, CA, U.S.A.) in a humidified incubator with 5% CO_2_ at 37°C. A library of 12000 compounds (synthetic and natural) was earlier screened for anticancer candidates capable of abrogating mortalin–p53 interactions resulting in nuclear enrichment of p53 and a shift in mortalin staining from perinuclear (concentrated around the nuclear membrane; typical of cancer cells) to pan cytoplasmic (widely distributed in the cell cytoplasm; typical of normal cells) type [31]. Out of 12000 compounds screened, only 6 were finally selected and further tested for their anticancer potential in *in vitro* and *in vivo* analyses. Two out of six (CL-44, named Mortaparib; and CL-49, named Mortaparib^Plus^) showed considerable anticancer potential as they inhibit mortalin and PARP1 [31,32]. In the present study, binding affinity of CL-44/Mortaparib (5-[1-(4-methoxyphenyl)(1,2,3,4-tetraazol-5-yl)]-4-phenylpyrimidine-2-ylamine) and CL-49/Mortaparib^Plus^ (4-[(1E)-2-(2-phenylindol-3-yl)-1-azavinyl]-1,2,4-triazole) to cell surface receptor proteins (ACE2 and TMPRSS2) was investigated using *in silico* molecular modeling approaches. Effects on mRNA and protein expression were determined by RT-qPCR and Western blotting, respectively.

### Computational analyses

#### Preparation of protein and ligand structures

To analyze the effect of Mortaparib and Mortaparib^Plus^ against the SARS-CoV-2 infection and multiplication, three crucial proteins were targeted, namely, M^pro^, TMPRSS2, and ACE2. The 3D structure of M^pro^ and ACE2 was retrieved from Protein Data Bank (PDB) having PDB ID: 6LU7 [33] and 6LZG [34], respectively. Since the PDB structure is not yet available for TMPRSS2, the homology modeled TMPRSS2 structure (prepared using serine protease hepsin (5ce.1.1) as template) was retrieved from the Swiss model repository (O15393). All these structures were further prepared for docking using the protein preparation wizard of the maestro from Schrodinger suite [35,36]. The main steps for the preparation of structures involved the removal of water molecules, the addition of missing hydrogens (H-bond) and disulfide (SS-bond) bonds, filling of missing side chains, and optimization of added hydrogens. Then OPLS3e force field was used for restrained minimization until the average root mean square deviation (RMSD) of the non-hydrogen atoms converged to 0.30 Å [37]. Further, the structure of Mortaparib and Mortaparib^Plus^ was sketched using Marvin sketch software [38]. The sketched structures were prepared for molecular docking using Ligprep module of Schrodinger suite [39]. Ligand preparation steps mainly included energy minimization using the OPLS3e force field, generation of all the ionization states at pH 7.0 ± 2, desalting of ligand, and possible tautomer and stereoisomers generation.

#### Grid generation and molecular docking

After preparation of the protein and ligand structures, the grid was generated at the active site of all the proteins to dock both ligands. In case of M^pro^, a grid of 20 Å edge was generated around Phe^140^, Asn^142^, Gly^143^, His^164^ and Glu^166^, as these residues make polar contacts with the already known peptide-like inhibitor N3 in the crystallized structure of M^pro^ (PDB ID: 6LU7) [33]. In the case of TMPRSS2, the grid was generated by choosing the three main catalytic residues namely, His^296^, Asp^345^, and Ser^441^ [40]. While for ACE2, it was found that in the native crystal structure, Ser^19^, Gln^24^, Lys^34^, Glu^35^, Asp^38^, and Gln^42^ of ACE2 were involved in the hydrogen bonding with receptor binding domain of Spike protein [41]. So, the grid was generated around these interacting residues. Subsequently, the extra precision flexible docking was performed for all the prepared systems using the Glide module of Schrodinger suite [42].

#### Molecular dynamics simulations of the docked systems

The best-docked poses of the protein–ligand complexes were further taken for molecular dynamics (MD) simulations using Desmond from the Schrodinger suite [36]. The MD simulation protocol used here has been described in detail in our previous study [43]. Each of the protein–ligand systems was solvated in TIP3P water model. Then the solvated systems were neutralized by adding appropriate number of counter ions (Na^+^/Cl^−^). Further energy of the systems was minimized by 100 ps Brownian MD at 10 K temperature in the NVT ensemble for removing any steric clashes. The minimized systems were equilibrated in seven steps using ‘Relax Model System Before Simulation’ in the Desmond MD GUI that performs the NPT/NVT equilibration. Then the equilibrated systems were subjected to 100 ns of the MD production run in NPT ensemble at 300 K temperature (Nose–Hoover chain thermostat), 1 atm pressure (Martyna–Tobias–Kelinbarostat), 2 fs time step, and recording interval of 20 ps.

The simulated systems were analyzed for the RMSD, Root mean square fluctuation (RMSF), Hydrogen bonds count, Radius of gyration (Rg), and Solvent accessible surface area (SASA) using simulation event analysis and simulation interaction diagram tool of Desmond module of Schrodinger. Finally, the MM/GBSA free energy was calculated for each protein–ligand system by extracting 100 structures from the simulation trajectory corresponding to 40–100 ns with an interval of 30 frames [39,44].

### Cell viability assay

Cytotoxicity of the Mortaparib and Mortaparib^Plus^ in human esophageal squamous carcinoma (T.Tn)-derived cells was determined by MTT (3-(4,5-dimethylthiazol-2-yl)-2,5-diphenyltetrazolium bromide) assay. A total of 4 × 10^3^ cells per well were plated in a 96-well plate, allowed to settle overnight, and treated with either Mortaparib or Mortaparib^Plus^. The control (DMSO) or treated cells were incubated for 48 h followed by the addition of 10 µl of phosphate-buffered saline (PBS) containing 5 mg/ml MTT (M6494, Life Technologies, Carlsbad, CA, U.S.A.), and further incubated for 4 h. Culture medium containing MTT was aspirated and replaced with DMSO to dissolve the formazan crystals. The plates were shaken for 5 min followed by measurement of optical density at 570 nm using Tecan bite M200 Pro microplate reader (Tecan Group Ltd., Mannedorf, Switzerland). Cell viability was calculated as a percentage against the control to identify their inhibitory concentration (IC) value using Microsoft Office 2016. Statistical significance was calculated by an unpaired *t* test of Microsoft Excel software (2016). Non-toxic dose (1 μM) of Mortaparib and Mortaparib^Plus^ were selected for further study.

### Reverse transcription quantitative PCR

A total of 2 × 10^5^ cells per well were plated in a six-well plate, allowed to settle overnight, followed by treatment with either Mortaparib or Mortaparib^Plus^. The control or treated cells were incubated at 37°C with 5% CO_2_. After 48 h, RNA was isolated from control and treated cells using the RNeasy Mini Kit (Qiagen, Stanford Valencia, CA, U.S.A.) following the manufacturer’s instructions. Equal amounts of RNA from each sample were reverse transcribed using the QuantiTect Reverse Transcription Kit (Qiagen, Tokyo, Japan). SYBR Select Master Mix was used to perform reverse transcription quantitative PCR (RT-qPCR) (Applied Biosystems, Life Technologies, Foster City, CA, U.S.A.). RT-qPCR was performed at 50°C for 2 min, 95°C for 10 min, followed by 40 cycles (denaturing at 95°C for 15 s, annealing at 60°C for 1 min, and extension at 72°C for 15 s). A melting curve was then created to evaluate the PCR amplification specification. To normalize the diversity in expression levels, the geometric mean of the housekeeping gene 18S was used as an internal control. Details of the primers are listed in Table 1.

**Table 1:** Primer sequences used for RT-qPCR

Gene (human)	Primer sequence (5′–3′)
*TMPRSS2* forward	GAGGACGAGAATCGGTGTGT
*TMPRSS2* reverse	TCCAGTCGTCTTGGCACA
*ACE2* forward	CATTGGAGCAAGTGTTGGATCTT
*ACE2* reverse	GAGCTAATGCATGCCATTCTCA
*18S* forward	CAGGGTTCGATTCCGTAGAG
*18S* reverse	CCTCCAGTGGATCCTCGTTA

### Western blotting

T.Tn cells (20 × 10^4^/well) were plated in a six-well plate, allowed to settle overnight, followed by treatment with Mortaparib or Mortaparib^Plus^. The control or treated cells were incubated at 37°C and 5% CO_2_. After 48 h, control and treated cells were harvested and washed twice with PBS, followed by lysis in RIPA buffer (Thermo Fisher Scientific, Rockford, IL, U.S.A.) containing complete protease inhibitor cocktail (Complete, Mini™; Roche Applied Science, Mannheim, Germany) on ice for 30 min. Lysates were centrifuged at 15000 rpm for 10 min, and the supernatant was collected for Western blotting with the indicated antibodies. The bicinchoninic acid assay (BCA) was used to determine the protein contents in cell lysates (Thermo Fisher Scientific, U.S.A.). The lysates containing 10–20 μg protein were separated on 8–10% SDS/polyacrylamide gel electrophoresis (SDS/PAGE) and transferred to a PVDF membrane (Millipore, Billerica, MA, U.S.A.) using wet transfer (Tank Blotting Cells system, Mini Trans-Blot Cell] (Bio-Rad, California, U.S.A.). Membranes were blocked with 3% BSA (WAKO, Japan) at room temperature for 1 h. Primary antibodies specific for target proteins (1–3 μg/ml) were used to probe the blocked membranes, including TMPRSS2 (ab92323) and ACE2 (ab15348) (Abcam, Cambridge, U.K.). Anti-rabbit IgG (Santa Cruz Biotechnology, CA, U.S.A.) conjugated to horseradish peroxidase was used as a secondary antibody, and the blots were developed using enhanced chemiluminescence (ECL) (GE Healthcare, Buckinghamshire, U.K.). As an internal loading control, Direct-Blot HRP anti-β-actin antibody (Lot303080) (BioLegend CNS, Inc, San Diego, California, United States) was used. The protein signals were quantified using ImageJ 1.46 (National Institutes of Health, Bethesda (NIH), MD, U.S.A.) software.

### Immunocytochemistry

T.Tn cells (4 × 10^4^/well) were plated on 18-mm glass coverslips placed in 12-well culture dishes (TPP, Trasadingen, Switzerland). The following day, cells were treated with Mortaparib- or Mortaparib^plus^-supplemented medium and incubated for 48 h. Cells were then washed with PBS twice and fixed in methanol:acetone (1:1) at 4°C for 5–10 min. After removing the fixation solution, the cells were washed with PBS thrice and then permeabilized in PBS with 0.1% Triton X-100 (PBST) for 10 min. Cells were blocked with 2% BSA (WAKO, Japan) at room temperature for 1 h. Bovine serum albumin (2%) containing primary antibodies (1–2 μg/ml) (TMPRSS2 (ab109131) and ACE2 (ab15348) (Abcam, Cambridge, U.K.) was used and incubated overnight. Secondary antibodies (1–2 μg/ml) were applied as specified, conjugated with Texas RED (Amersham Biosciences, Buckinghamshire, U.K.) or FITC, Alexa-488, or Alexa-594 (Molecular Probes, Eugene, OR, U.S.A.). Nuclei were stained with Hoechst (Invitrogen, Molecular Probes, Eugene, OR, U.S.A.). Cells were observed under a Zeiss Axiovert 200 M microscope (Carl Zeiss, Tokyo, Japan) using 40× objective lens, and the results were analyzed using AxioVision 4.6 software. The fluorescence signals were quantified using ImageJ 1.46 (NIH, Bethesda, MD, U.S.A.) software.

### Antiviral activity assay

The assay was done in a 96-well plate in triplicates. A total of 1 × 10^4^ Vero E6 cells (kidney epithelial cells from *Cercopithecus aethiops*, ATCC) were plated per well and incubated at 37°C overnight for the monolayer formation. Cells were incubated with the culture medium containing Mortaparib/Mortaparib^Plus^ (dissolved in DMSO) at a non-toxic concentration (as determined by independent cytotoxicity assay). This was followed by the addition of SARS-CoV-2 (USA-WA1/2020 strain) at a 0.01 multiplicity of infection. Control cells were incubated with culture medium with 0.5% DMSO. Culture supernatant was harvested post-24 and -48 h from the plates incubated at 37°C. Viral RNA was isolated from 100 µl cell culture supernatant using PureLink Viral RNA/DNA Mini Kit (Invitrogen). cDNA was prepared using ImProm-II Reverse Transcription System (Promega). Real-time PCR was performed in QuantStudio 6 Flex Real-Time PCR System using TB Green Premix Ex Taq II (TaKaRa). The following primers were used to quantify the viral RNA levels: (a) Envelope (E) primers 5′-ACAGGTACGTTAATAGTTAATAGCGT-3′ (forward), 5′-ATATTGCAGCAGTACGCACACA-3′ (reverse). The *C*_t_ values for E gene sequence detection were determined and used for calculating the percent virus inhibition with respect to the control.

### Statistical analysis

The mean and standard deviation of data from three or more experiments were calculated. The degree of significance between the control and experimental samples was determined using an unpaired *t* test (GraphPad Prism Software, San Diego, CA, U.S.A.). Statistical significance was defined as non-significant (^ns^*P*-value >0.05), significant (*P-value ≤0.05), very significant (**P-value ≤0.01), highly significant (***P-value ≤0.001), and extremely significant (****P-value ≤0.0001).

## Results and discussion

### Computational analyses of Mortaparib and Mortaparib^Plus^ as potential inhibitors of TMPRSS2 and ACE2

**Figure 1:**
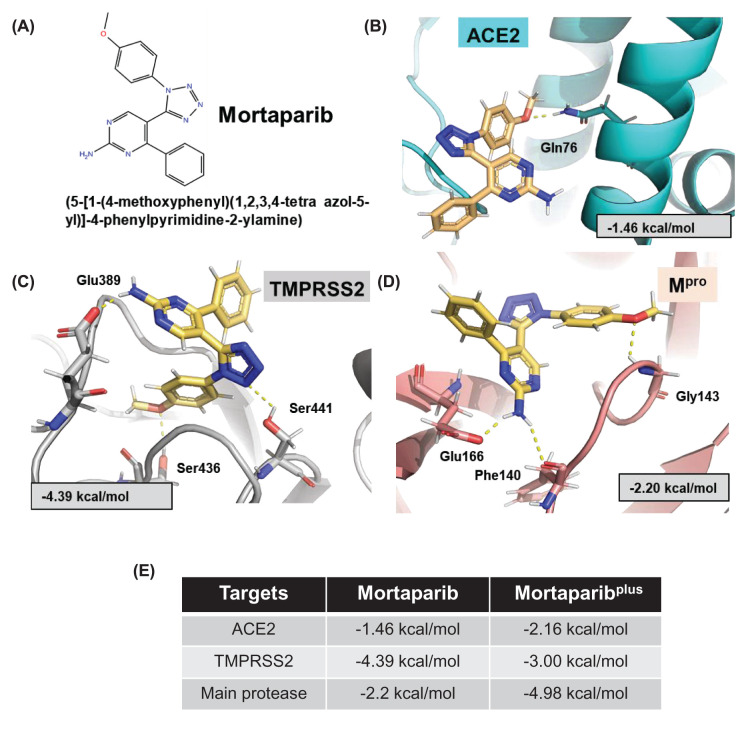
The docked pose of ligands with the target proteins (**A**) 2D structure of Mortaparib. (**B**) The best docked pose, the docking score, and hydrogen bond interactions of Mortaparib with ACE2, (**C**) TMPRSS2, (**D**) M^pro^. (**E**) The docking score of Mortaparib and Mortaparib^Plus^ against the target proteins.

**Figure 2:**
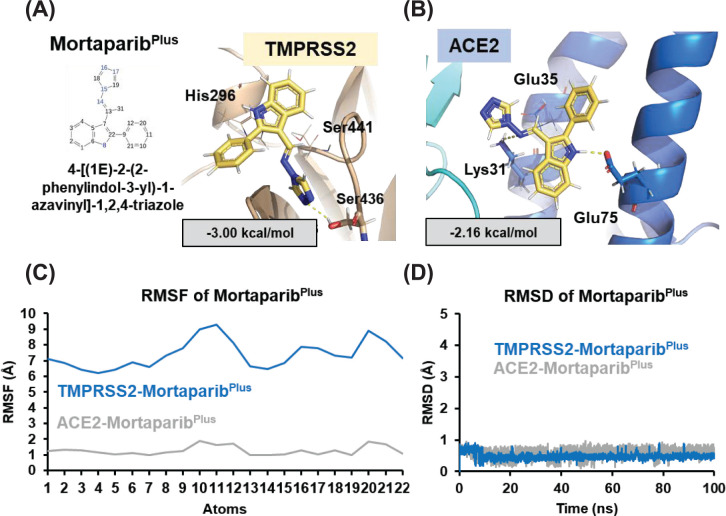
Analyses of the structural interactions of Mortaparib^Plus^ with the host cell proteins (**A**) Best docked pose Mortaparib^Plus^-TMPRSS2. (**B**) Best docked pose of Mortaparib^Plus^-ACE2. (**C**) RMSF of Mortaparib^Plus^ residues throughout the simulation of 100 ns when bound with TMPRSS2 and ACE2. (**D**) The RMSD of Mortaparib^Plus^ when bound with TMPRSS2 and ACE2 showing a stable binding.

**Figure 3:**
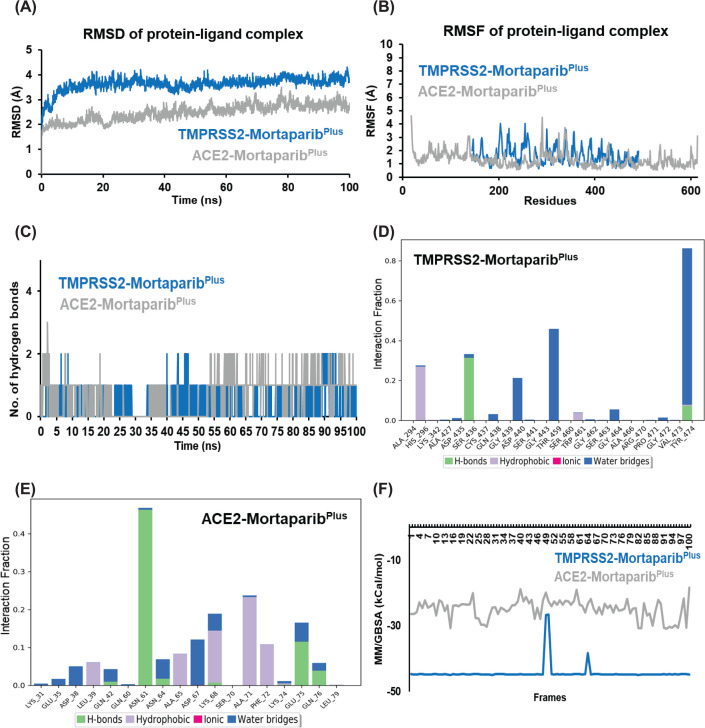
The analysis of the MD simulations of Mortaparib^Plus^ with the host cell proteins (**A**) The RMSD of protein–ligand complexes showing overall stability of the complexes in the dynamic solvated environment. (**B**) The RMSF of the amino acid residues of the proteins when bound with Mortaparib^Plus^. (**C**) The number of hydrogen bonds formed throughout the simulation trajectory of Mortaparib^Plus^ bound proteins showing that Mortaparib^Plus^ had better interaction with TMPRSS2 in comparison to ACE2. (**D**) The interaction fraction of the amino acid residues TMPRSS2 and **(E**) ACE2 with Mortaparib^Plus^ throughout the simulation. (**F**) The MM/GBSA free energy binding of Mortaparib^Plus^ with the proteins. Mortaparib^Plus^ had a higher binding affinity for TMPRSS2 than ACE2.

We used glide flexible molecular docking and examined if Mortaparib or Mortaparib^Plus^ (structure shown in Figures 1A and 2A, respectively) could interact with TMPRSS2 and ACE2 and act as their inhibitors. The docking results showed that both compounds could bind to the active pocket of the targeted proteins. However, the docking score of Mortaparib with ACE2 was considerably less (−1.46 kcal/mol) in comparison to TMPRSS2 (−4.39 kcal/mol). In the best binding pose, it showed hydrogen bonding with Gln^76^ of ACE2 and Ser^441^ (catalytic residue of TMPRSS2) and Ser^436^ of TMPRSS2 (Figure 1B,C). When these docked complexes were subjected to MD simulations to check the binding stability and consistency of the crucial interactions, Mortaparib could not interact stably with any of these two targets. It came out of the binding site within 50 ns of the simulation run. On the other hand, Mortaparib^Plus^ exhibited comparable docking score with TMPRSS2 (−3.00 kcal/mol) and ACE2 (−2.16 kcal/mol). In case of TMPRSS2, Mortaparib^Plus^ showed hydrogen bonding with Ser^436^, and polar interactions with two main catalytic residues His^296^ and Ser^441^ (Figure 2A). In case of ACE2–Mortaparib^Plus^ interactions, ACE2 residues (Lys^31^ and Glu^75^) that interact with viral Spike protein were found to be involved in hydrogen bonding. Glu^35^ also showed columbic interactions with Mortaparib^Plus^ in the best docked pose (Figure 2B). The docking score as well as binding characteristics (polar and non-polar interactions) for all the complexes are listed in Table 2. These docked complexes were also taken further for MD simulation of 100 ns to investigate the stability and dynamic behavior of the proteins when bound to Mortaparib^Plus^. When MD trajectories were examined, it was found that Mortaparib^Plus^ stayed at the binding site of both the proteins (Figure 2C). The bound ligand did not show fluctuations during the simulations, as shown in the RMSD plot (Figure 2D). The trajectories of protein and ligand complexes were also analyzed for investigating deviations in the overall structure. The RMSD plot showed structural stability without any significant deviation for all the complexes (Figure 3A). Similarly, in RMSF, no major fluctuation was seen throughout the duration of simulation (Figure 3B). Analysis of hydrogen bonds for each protein–ligand complex revealed that on an average Mortaparib^Plus^ made 0.38 ± 0.57 hydrogen bonds with TMPRSS2 and 0.65 ± 0.53 with ACE2 (Figure 3C). When significant fraction of interaction (interaction fraction > 30% of the simulation time) was calculated for the entire course of simulation, it was found that in case of TMPRSS2, two residues (Tyr^459^ and Tyr^474^) made significant water-based interactions with Mortaparib^Plus^, but none of the main catalytic residues (His^196^, Asp^345^, and Ser^441^) were involved in major interactions (Figure 3D). Similarly, in case of ACE2, only one residue (Asn^61^) was involved in significant interaction, while none of the residues of ACE2 that make hydrogen bonds with the Spike protein (Ser^19^, Gln^24^, Lys^34^, Glu^35^, Asp^38^, and Gln^42^) significantly interacted with Mortaparib^Plus^ (Figure 3E) [41]. Further, Rg and SASA of Mortaparib^Plus^ bound with different proteins was also calculated. All the results showed that Mortaparib^Plus^ could bind stably with both the examined proteins (listed in Table 3). Finally, the MM/GBSA free energy calculations to investigate the binding affinity of Mortaparib^Plus^ for the individual proteins revealed that Mortaparib^Plus^ had higher affinity for TMPRSS2 (−44 ± 2.62 kcal/mol) than ACE2 (−23.74 ± 10.89 kcal/mol) (Figure 3F). These computational analyses suggested that (i) Mortaparib^Plus^, but not Mortaparib, could bind stably with ACE2 and TMPRSS2 and (ii) the interactions were significantly stronger with TMPRSS2.

**Table 2:** Residues of M^pro^, ACE2, and TMPRSS2 interacting in the best docked pose and the docking score of the complexes

Complex	Molecular docking (kcal/mol)	Types of interactions and residues involved (pre-MD simulations)	
		H-bonds	Hydrophobic, polar, and π–π stacking
M^pro^–Mortaparib^Plus^	−4.98	His^164^	His^41^, Met^49^, Pro^52^, Tyr^54^, Phe^140^, Leu^141^, Asn^142^, Ser^144^, Cys^145^, His^163^, Met^165^, Glu^166^, Asp^187^, Arg^188^, Gln^189^, Thr^190^, Gln^192^
TMPRSS2– Mortaparib^Plus^	−3.00	Ser^436^	Val^280^, His^296^, Lys^342^, Cys^437^, Gln^438^, Gly^439^, Ser^441^, Thr^459^, Ser^460^, Trp^461^, Gly^462^, Ser^463^, Gly^464^, Cys^465^
ACE2– Mortaparib^Plus^	−2.16	Lys^31^, Glu^75^	Glu^35^, Leu^39^, Lys^68^, Phe^72^, Gln^76^, Leu^79^

**Table 3:** MD trajectory analysis of RMSD, RMSF, hydrogen bond counts, MM/GBSA free energy, Rg, and SASA for the simulated systems

Properties	Protein–ligand complex			Ligand		
	M^pro^– Mortaparib^Plus^	TMPRSS2– Mortaparib^Plus^	ACE2– Mortaparib^Plus^	Mortaparib^Plus^ with M^pro^	Mortaparib^Plus^ with TMPRSS2	Mortaparib^Plus^ with ACE2
RMSD (Angstrom)	2.29 ± 0.14	3.63 ± 0.27	2.48 ± 0.32	0.57 ± 0.07	0.48 ± 0.09	0.61 ± 0.13
RMSF (Angstrom)	1.26 ± 0.59	1.62 ± 0.68	1.28 ± 0.56	1.32 ± 0.32	1.27 ± 0.29	7.38 ± 0.89
Hydrogen bonds count (protein–ligand complex)	0.99 ± 0.29	0.38 ± 0.57	0.65 ± 0.53	-	-	-
MM/GBSA free binding energy	−47.37 ± 0.07	−44.07 ± 2.62	−23.74 ± 10.89	-	-	-
Rg (Angstrom)	-	-	-	3.70 ± 0.02	3.70 ± 0.02	3.71 ± 0.02
SASA (A^2^)	-	-	-	114.34 ± 16.26	109.03 ± 23.87	282.96 ± 28.68

### TMPRSS2 and ACE2 expression analyses in cells treated with Mortaparib or Mortaparib^Plus^

**Figure 4:**
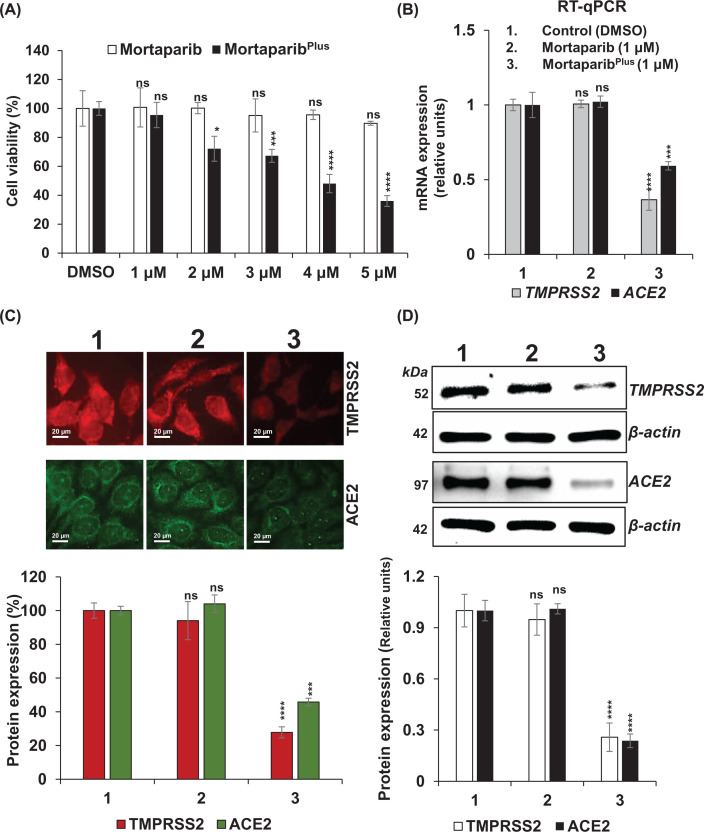
Mortaparib^Plus^, but not Mortaparib, caused down-regulation of TMPRSS2 and ACE2 expression (**A**) Dose-dependent cytotoxicity of the two compounds, as determined by MTT assay is shown. (**B**) TMPRSS2 and ACE2 mRNA expressions in control and treated (non-toxic dose of Mortaparib and Mortaparib^Plus)^ cells. (**C**) Immunostaining and (**D**) Western blotting showing the expression of TMPRSS2 and ACE2 in control and treated cells. Data were normalized against control and plotted as fold difference. Each data set represented the mean SD of at least three independent experiments. Statistical significance was defined as non-significant (nsp-value > 0.05), significant (*P-value ≤ 0.05), highly significant (***P-value ≤ 0.001) and extremely significant (****P-value ≤ 0.0001)

In the MTT-based cell viability assay, Mortaparib and Mortaparib^Plus^ showed dose-dependent toxicity in the T.Tn cells. Mortaparib^Plus^ was relatively stronger than Mortaparib (Figure 4A). Similar cytotoxicity has been reported earlier in a variety of other cancer cell types [31,32]. In the present study, we selected non-toxic dose (1 μM) for both the compounds. T.Tn cells were treated with 1 μM (Mortaparib or Mortaparib^Plus^) for 48 h followed by the expression analysis of the target proteins. As shown in Figure 4B, Mortaparib^Plus^-treated cells showed 50–60% down-regulation in both ACE2 and TMPRSS2 mRNA as determined by RT-qPCR. In contrast, Mortaparib-treated cells showed no effect. Protein expression as detected by immunostaining as well as Western blotting showed reduction in TMPRSS2 and ACE2 proteins in Mortaparib^Plus^, but not in Mortaparib, treated cells (Figure 4C,D). The raw images of the Western blot showing the changes in the expression of TMPRSS2 and ACE2 in response to treatment with Mortaparib^Plus^ are also presented in Supplementary Figures S1 and S2.

### Mortaparib and Mortaparib^Plus^ as a potential inhibitor of viral protein M^pro^

**Figure 5:**
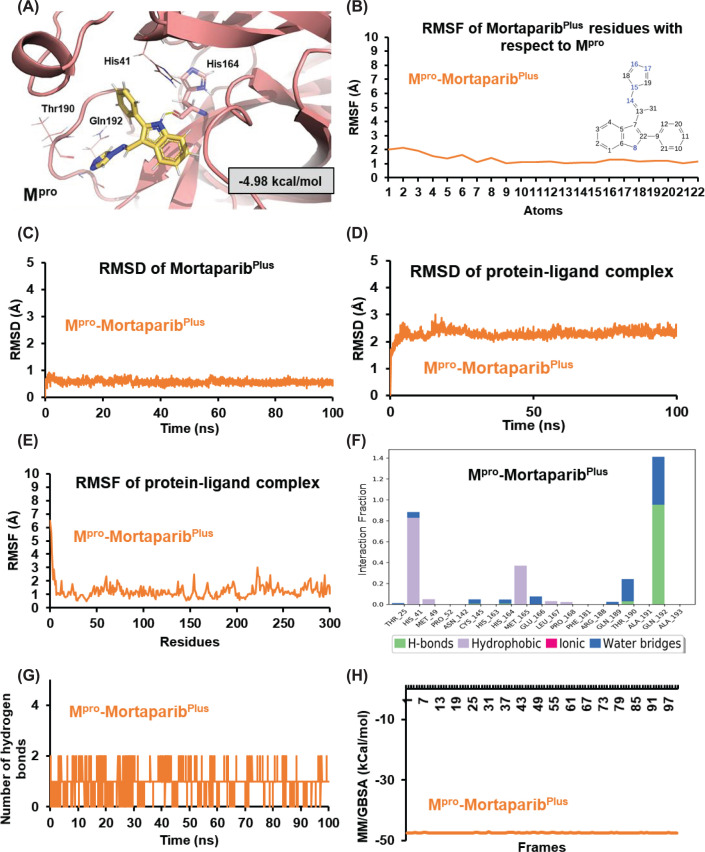
Analyses of the structural interactions after docking and MD simulations of Mortaparib^Plus^ with the viral protein M^pro^ (**A**) Best docked pose of Mortaparib^Plus^–M^pro^. (**B**) The RMSF of Mortaparib^Plus^. (**C**) The RMSD of Mortaparib^Plus^ when bound with M^pro^ showing a stable binding. (**D**) The RMSD of protein–ligand complex showing overall stability in the aqueous environment. (**E**) The RMSF of the amino acid residues of M^pro^ when bound with Mortaparib^Plus^. (**F**) The interaction fraction of the amino acid residues of M^pro^ with Mortaparib^Plus^. (**G**) The count of hydrogen bonds throughout the simulation between Mortaparib^Plus^ and M^pro^. (**H**) The MM/GBSA free energy of binding of Mortaparib^Plus^ with M^pro^ showing higher affinity for M^pro^ than both TMPRSS2 and ACE2.

We next investigated the binding ability of the Mortaparib and Mortaparib^Plus^ to viral M^pro^ protein. The compounds were docked at the active site of the M^pro^. The docking score of Mortaparib was −2.20 kcal/mol and in the best binding pose it was making hydrogen bonds with Phe^140^, Gly^143^, and Glu^166^ of M^pro^ (Figure 1D). The comparsion of docking score of Mortaparib and Mortaparib^plus^ against all the three targets (ACE2, TMPRSS2 and M^pro^) have been shown in Figure 1E. However, when the complex was simulated for 100 ns it was found that Mortaparib was not stable at the binding pocket of M^pro^, and hence was not studied further. The docking score of Mortaparib^Plus^ was considerably higher at the binding site of M^pro^ (−4.98 kcal/mol) in comparison to the host cell surface proteins—TMPRSS2 and ACE2. In the case of M^pro^–Mortaparib^Plus^ best docking pose, His^164^ was making hydrogen bonds while the other crucial catalytic residues— His^41^ and Thr^190^ of M^pro^ were involved in other polar and hydrophobic interactions (Figure 5A). The docking score and details of interacting residues are listed in Table 2. The docked complex was then simulated in water for 100 ns to investigate the stability and dynamic behavior of the M^pro^–Mortaparib^Plus^ complex. Mortaparib^Plus^ was found to bind stably at the active site in M^pro^ throughout the simulation (Figure 5B). RMSD plot of Mortaparib^Plus^ also showed its stability within the M^pro^ pocket (Figure 5C). The overall structure of the bound protein also did not show any significant deviations (Figure 5D). Similarly, in RMSF analyses, no major fluctuations were seen (Figure 5E). Next, the interaction fraction time of each active site residue with the Mortaparib^Plus^ was calculated for the simulated trajectory, which showed that the three main catalytic and conserved residues (His^41^, Met^165^, and Gln^192^) of M^pro^ made significant interactions (i.e., >30% of the simulation time) with Mortaparib^Plus^ (Figure 5F). The average number of hydrogen bonds that Mortaparib^Plus^ made with M^pro^ during the MD run was 0.99 ± 0.29 (Figure 5G). The characteristic values of Mortaparib^Plus^ in terms of RMSD, RMSF, Rg, and SASA are listed in Table 3. Finally, the MM/GBSA free energy of Mortaparib^Plus^ binding to M^pro^ showed a strong affinity (−47.37 ± 0.07 kcal/mol) (Figure 5H), which was much higher than that for the host cell proteins, TMPRSS2 and ACE2. These data indicated that Mortaparib^Plus^ may possess the potential to inhibit the activity of M^pro^ as well. Taken together, these *in silico* data suggested that Mortaparib^Plus^ may inhibit virus infection by its impact on host cell receptors as well as virus proteins.

### Antiviral activity of Mortaparib and Mortaparib^Plus^

We then investigated the effect of Mortaparib and Mortaparib^Plus^ on SARS-CoV-2 replication. Cells treated with a known inhibitor, Remdesivir (10 μM), showed ∼95% inhibition of virus replication based on E-gene RT-PCR at 24 h post-infection. In several parallel independent assays, we detected 10–15 and 30–50% inhibition of virus replication (as determined by qPCR of virus E-gene sequence) in cells treated with 0.5 and 1.0 μM Mortaparib^Plus^, respectively. Intriguingly, Mortaparib-treated cells also showed equivalent inhibition (15–25 and 30–50% with 0.5 and 1.0 μM, respectively).

## Discussion

Host cell surface receptor protein, TMPRSS2, is known to interact with the SARS-CoV-2 virus and cause splicing of the viral S protein into S1 and S2 [20]. The former is essential for the virus-to-host fusion, while the latter interacts with the host cell receptor ACE2 and helps in internalization. Both TMPRSS2 and ACE2 are known to be enriched in lung, heart, kidney and intestinal endothelia, and operate as the first line of defense against the foreign pathogens. TMPRSS2 has been suggested to play a significant role in several physiological and pathological mechanisms involving internalization (such as the facilitation of sperm function) or inflammatory response (such as airway defense) [25–27]. It has been shown to be pathologically up-regulated in the prostate and colon cancer cells [40,45]. ACE2, on the other hand, is commonly known to physiologically catalyze the hydrolysis of angiotensin II to angiotensin and contribute to vasodilatation [28,29], and to pathologically facilitate SARS-CoV-2 and similar infections [46]. Thus, while the TMPRSS2 and ACE2 proteins are some of the major targets of the coronaviruses, their excessive suppression by targeted therapies may result into the appearance of unprecedented collateral adverse effects such as the male infertility, frequent respiratory infections, and hypertension [47,48]. Another crucial target to fight against SARS-CoV-2 is M^pro^, which cleaves the polyprotein into functional proteins to help in its replication process [49]. Inhibition of its activity can stop the production of new virus particles and transmission. Its similarity with previously reported BatCoV RaTG13 M^pro^ (99%) and SARS-CoV M^pro^ (96%) makes it an important target, and therefore identification of potent compounds against M^pro^ can help in the pan-coronavirus inhibition [49].

By computational and *in vitro* experimental assays, we investigated the impact of Mortaparib and Mortaparib^Plus^ on host cell receptor proteins, ACE2 and TMPRSS2 and viral protein, M^pro^. Mortaparib^Plus^ showed higher affinity towards TMPRSS2 in comparison to ACE2 as observed from molecular docking and MM/GBSA free binding energy calculations. The difference between the average MM/GBSA binding free energy of Mortaparib^Plus^ against ACE2 (−23.74 kcal/mol) and TMPRSS2 (−44.07 kcal/mol) was almost double, and therefore it was predicted to block TMPRSS2 more strongly than ACE2. Taken together with the dispensable role of TMPRSS2, Mortaparib^Plus^ is predicted to be a safer candidate therapeutic alternative for SARS-CoV-2 [50–52]. Furthermore, it was also predicted to inhibit viral M^pro^ that is crucial for viral replication in the host cells (Figure 4 and Tables 2 and 3). Of the three protein targets studied here, Mortaparib^Plus^ had the comparatively highest binding affinity towards the M^pro^ (−47.37 kcal/mol), followed by TMPRSS2 (−44.07 kcal/mol), and ACE2 (−23.74 kcal/mol). On the other hand, binding of Mortaparib was not stable with any of these protein targets. Although the MM/GBSA binding free energy showed a decent binding affinity of the Mortaparib^plus^ with the target proteins, they may not be indicating the exact experimental values but are correlative and in reference to each other. The more negative values indicate higher affinity of the ligands for the target proteins. Furthermore, the computational results of the TMPRSS2 are based on the homology model as the experimental structure is not yet available, although the modeled structure is highly likely to be close to experimental one, but it may deviate. Furthermore, expression assays revealed down-regulation of TMPRSS2 and ACE2 in Mortaprib^Plus^, but not Mortaparib, treated cells. However, in antiviral assays both Mortaparib^Plus^ and Mortaparib caused comparable inhibition of viral replication. These data suggested that the two compounds possess multimodal anti-SARS-CoV-2 activity. Whereas Mortaparib^Plus^ causes inhibition of host cell receptors—ACE2 and TMPRSS2, and the virus protein—M^pro^, Mortaparib involves independent mechanisms. Further experimental and clinical studies are warranted to resolve these mechanisms, safety of these compounds to consider their recruitment for COVID-19 treatment.

## Conclusion

In the present study, we have investigated the anti-COVID-19 activity of Mortaparib and Mortaparib^Plus^ against three target proteins, namely ACE2, TMPRSS2, and M^pro^ using bioinformatics approaches and cell-based assays. The computational study suggested that Mortaparib^Plus^ has the highest binding affinity towards the catalytic site of M^pro^ followed by TMPRSS2 and then ACE2. Mortaparib showed poor affinity. *In vitro* experiments also showed that Mortaparib^Plus^, but not Mortaparib, caused down-regulation of TMPRSS2 and ACE2 mRNA and proteins. However, both compounds caused inhibition of viral replication in cell-based assays suggesting their multimodal anti-SARS-CoV-2 activities. Further studies are required to resolve multimodal anti-COVID-19 potential of these compounds.

## Supplementary Material

Supplementary Figures S1-S2

## Data Availability

The authors confirm that the data supporting the findings of the present study are available within the article and/or its supplementary materials. All the structural data used in the study were directly retrieved from the PDB (https://www.rcsb.org/) or Swiss Homology Repository (https://swissmodel.expasy.org/repository). None of the structures have been generated newly or modified.

## Abbreviations

ACE2: angiotensin-converting enzyme 2
COVID-19: coronavirus disease 2019
MD: molecular dynamics
M^pro^: main protease
MTT: 3-(4,5-dimethylthiazol-2-yl)-2,5-diphenyltetrazolium bromide
PARP-1: Poly [ADP-ribose] polymerase 1
PBS: phosphate-buffered saline
PDB: Protein Data Bank
Rg: radius of gyration
RMSD: root mean square deviation
RMSF: root mean square fluctuation
RT-qPCR: reverse transcription quantitative PCR
SARS-CoV-2: severe acute respiratory syndrome coronavirus 2
SASA: solvent accessible surface area
TMPRSS2: transmembrane protease serine 2
