# Supplementary material for: Computational and in vitro experimental analyses of the anti-COVID-19 potential of Mortaparib and MortaparibPlus
Source: Biosci Rep. 2021 Oct 14;41(10):BSR20212156. doi: 10.1042/BSR20212156 (PMC8527209; doi:10.1042/BSR20212156)
Supplement: Supplementary Figures S1-S2 [file BSR-2021-2156_supp.pdf]

Supplementary Figures

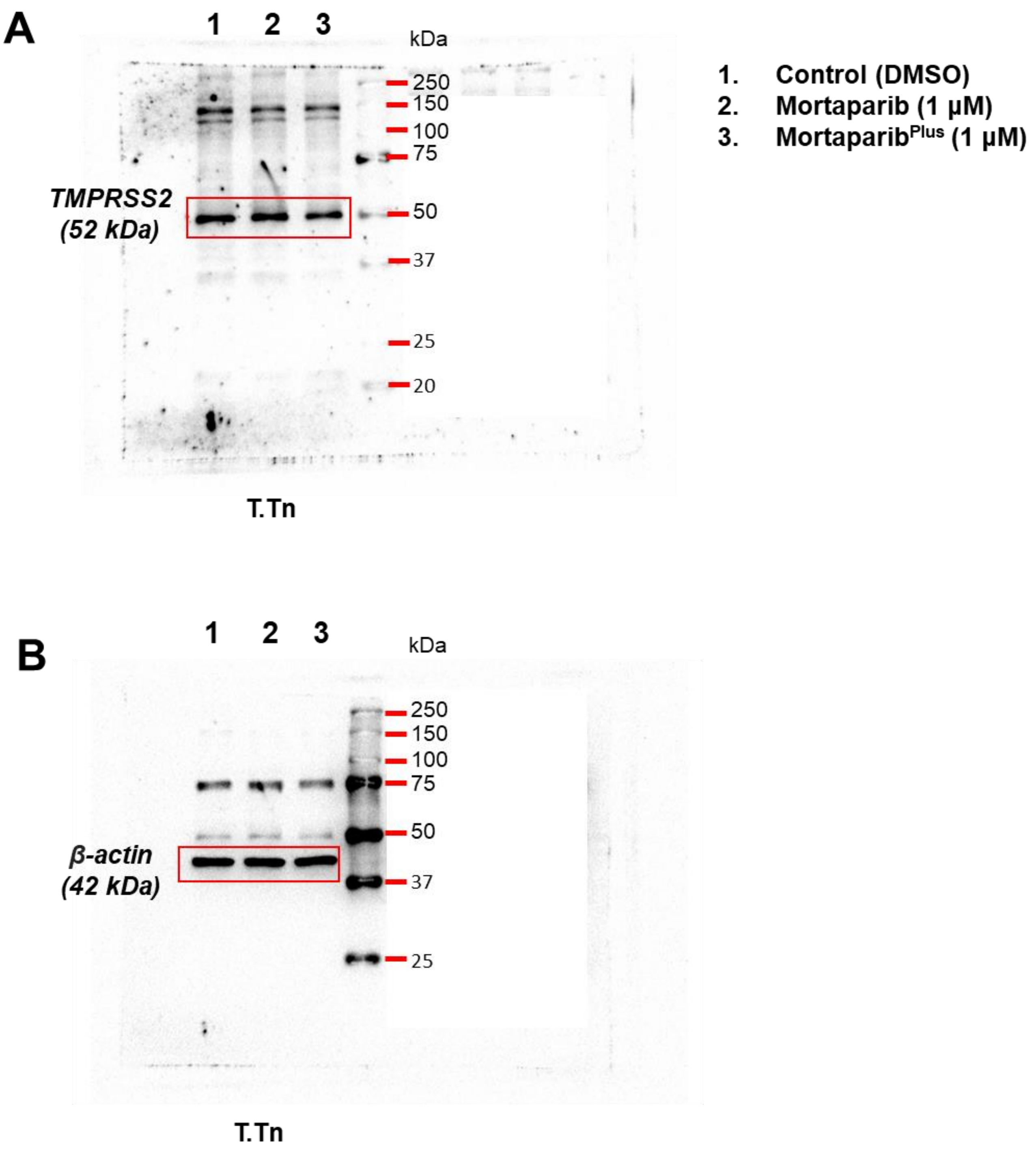

Figure S1. Full image of western blot showing the expression of the (A) TMPRSS2 (B) β-actin (loading control) in control and treated cells.

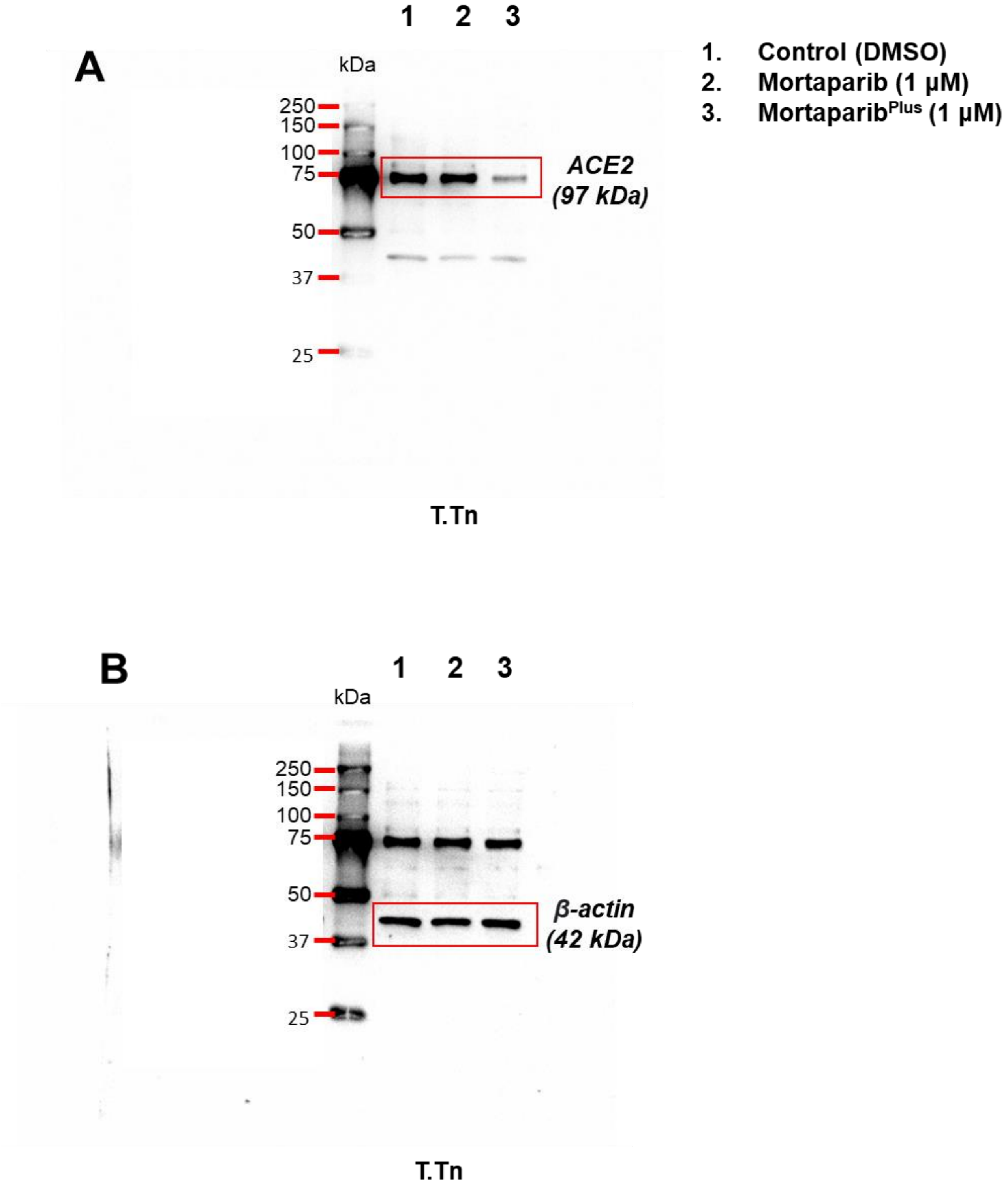

Figure S2. Full image of western blot showing the expression of the (A) ACE2 and (B) β-actin (loading control) in control and treated cells.
